# Family care for persons with severe mental illness: experiences and perspectives of caregivers in Uganda

**DOI:** 10.1186/s13033-021-00470-2

**Published:** 2021-05-20

**Authors:** F. Verity, A. Turiho, B. B. Mutamba, D. Cappo

**Affiliations:** 1 College of Human and Health Sciences, Singleton, Swansea University, Wales, SA28PP UK; 2 Makerere University College of Health Sciences, Kampala, Uganda; 3 Butabika National Referral Mental Hospital, Kampala, Uganda; 4 YouBelong, Kampala, Uganda

**Keywords:** Family care, Severe mental illness, Hospital discharge, Community health, Uganda

## Abstract

**Background:**

In low-income settings with limited social protection supports, by necessity, families are a key resource for care and support. Paradoxically, the quality of family care for people living with Severe Mental Illness (PLSMI) has been linked to support for recovery, hospital overstay and preventable hospital readmissions. This study explored the care experiences of family members of PLSMI with patients at the national mental hospital in Kampala, Uganda, a low income country. This study was undertaken to inform the development of YouBelongHome (YBH), a community mental health intervention implemented by YouBelong Uganda (YBU), a registered NGO in Uganda.

**Methods:**

Qualitative data was analysed from 10 focus groups with carers of ready to discharge patients on convalescent wards in Butabika National Referral Mental Hospital (BNRMH), Kampala. This is a subset of data from a mixed methods baseline study for YouBelong Uganda, undertaken in 2017 to explore hospital readmissions and community supports for PLSMI from the Wakiso and Kampala districts, Uganda.

**Results:**

Three interrelated themes emerge in the qualitative analysis: a range of direct, practical care provided by the caregiver of the PLSMI, emotional family dynamics, and the social and cultural context of care. The family care giving role is multidimensional, challenging, and changing. It includes protection of the PLSMI from harm and abuse, in the context of stigma and discrimination, and challenging behaviours that may result from poor access to and use of evidence-based medicines. There is reliance on traditional healers and faith healers reflecting alternative belief systems and health seeking behaviour rather than medicalised care. Transport to attend health facilities impedes access to help outside the family care system. Underpinning these experiences is the impact of low economic resources.

**Conclusions:**

Family support can be a key resource and an active agent in mental health recovery for PLSMI in Uganda. Implementing practical family-oriented mental health interventions necessitates a culturally aware practice. This should be based in understandings of dynamic family relationships, cultural understanding of severe mental illness that places it in a spiritual context, different family forms, caregiving practices and challenges as well as community attitudes. In the Ugandan context, limited (mental) health system infrastructure and access to medications and service access impediments, such as economic and transport barriers, accentuate these complexities.

## Background

In low-income countries (LIC) with limited social protection, families assume more care roles [1, 2]. Additionally, it is recognised that there are major challenges for family carers often framed in a language of carer burden or stress [2, 3]. The focus of this paper is a qualitative exploration of experiences and perspectives of family care for people with SMI in Uganda, a low-income country in East Africa. This data is a subset of a wider study exploring community-based interventions for people with SMIs, which was conducted to inform action to redress overstay and overcrowding at Butabika National Referral Mental Hospital (BNRMH), Kampala, Uganda. Hospital overstay has implications for realisation of peoples human rights and quality of life, mental health recovery and physical health [4, 5].

In recent theoretical work, Keating, Eales, Funk, Fast and Min theorise a family care domain from a life course perspective, drawing attention to understandings of family care as variously motivated by love, reciprocity or obligation [6, p.150]. They cogently distinguish care as tasks and care as being in relationship [6, p.149]. Within this family care domain, life course events and transitions will influence care needs, experiences in and of care, resources and care supports [6]. In respect to care for people living with SMI in low-income countries, there are diverse factors that enable and jeopardise supportive community based and family care. These swirl around poverty, unemployment and low incomes, culture, beliefs and knowledge about mental illness, available primary care services, access to medication, as well as the nature of interpersonal care relationships [7,14].

Sfetcu and colleagues systematically reviewed published research (none from low-income countries) on post discharge variables and psychiatric hospital readmissions for the period between January 1990 and June 2014 [4]. Families were the focus of four of the 80 studies, and there was evidence of both the family unit to be supportive and unhelpful to mental health recovery [4]. Family stigma associated with mental illness, family functioning, levels of criticism and expressed emotion (EE) towards the person living with SMI and family support, variously shape care experiences. Culture can mediate these dynamic experiences. Some studies link expressed emotions (EE) of criticism and over involvement within the family unit to mental health relapse [15]. Azhar and Varma [16] in their Malaysian study of relapse and EE within families of 83 people living with schizophrenia, found that families reported low EE to their family member with schizophrenia, which the authors attribute to cultural factors. Jenkins and Karno in a comprehensive review of EE and culture write, Without a doubt, the nature and meaning of criticism and emotional overinvolvement are culturally specific [17, p19].

YouBelong Uganda (YBU), a Uganda registered non-government organisation established in 2016 to develop and support community options for people living with SMI in Uganda, has made family capability building a key component of its work. The care model developed by YBU has multi layers of influence [9]. It is informed by the ideas of the eighteenth century philosopher Johann Herder, and the twentieth century communitarian philosopher Charles Taylor. The latters approach is consistent with and builds on Herders view of the human person and society. It espouses the human person as relational in nature, and that human identity develops not in isolation, but within relationships with others in community. Within the YBH model, family is seen as a basic unit of care, and an active agent in relationships of care. This is not as a utilitarian measure using families as a fill in for a lack of care services in a resource poor setting, but as a basic level of relationships for the fulfilling of the human need for ongoing mutual love, care, security, and belonging. Community is also viewed as providing, at least in potential, and often in actuality, the relational dynamic necessary for the development of human identity and a sense of wellbeing and belonging. Set against this background, in this paper we explore family care and SMI in the Ugandan context, in order to understand better how mental health services can support people living with SMI within families and communities.

## Methods

The purpose of this paper is to explore the care experiences and perspectives of family members of a person living with SMI in Uganda, to inform the development of a community based mental health service delivery model called YouBelongHome. To explore this question, we have analysed the qualitative findings from focus groups with carers of ready to discharge patients on convalescent wards in BNRMH. The carers in this study live in either the Kampala district or the adjoining Wakiso district that spans a radius of up to 30 km from BNRMH. These two nearest local government districts are the main catchment areas for Butabika Hospital and account for over 50% of inpatient admissions.

This is a subset of data from a mixed qualitative and quantitative methods study undertaken in 2017. The wider study was commissioned by YBU and profiled the number of persons living with SMI hospitalised and or re-hospitalised at BNRMH; socio-demographic and illness characteristics, and wellbeing needs of those persons; and services and support for persons with SMI in families, communities and health facilities. Psychosis, Bipolar Affective Disorder, Substance Use Disorder, and Alcohol Use Disorder were the most common illness conditions among in-patients admitted on convalescent wards at BNRMH at the time of the study [18].

As part of the larger study, ten focus group discussions (FGDs) were held with adult caregivers of ready to discharge patients on convalescent wards in BNRMH, recruited based on information provided by the patients. The convalescent ward is where patients are placed when they are medically stabilised and approved for discharge, but family are not ready or not willing to have them returned, or lack the socio-economic resources to support return of the family member with SMI. In determining the sample size it was assumed that the sample of 10 groups (6080 participants) would be sufficient for theoretical saturation in view of the relative cultural/ linguistic homogeneity of the study population and the relatively few themes to explore based on objectives of the main study. Another consideration was the limited financial resources available for the study. Five of the 10 FGDs included participants from Kampala district and the other half were drawn from Wakiso. A maximum of 8 participants were targeted per FGD. After listing the patients according to districts, respective care givers were consecutively selected until the desired sample size of 80 (or 40 per district) was attained. Persons perceived by the concerned patients to be the most involved in their care were purposively selected to participate in the FGDs. The selected participants were facilitated to travel to attend the FGDs that were held in the lawns of the convalescent wards at BNRMH. Advance information was sent out to the selected FGD participants by telephone, specifying the date and time of the respective FGDs.

A trained qualitative interviewer moderated each group assisted by a trained note taker who made written notes and also audio recorded the proceedings to minimize loss of valuable details in the discussions. FGDs were conducted in Luganda, the main local dialect spoken in the two study districts. Each focus group lasted 6090 min and comprised 68 participants. The focus groups used a translated guide divided into sections based on objectives of the main study, with questions for participants about views and experiences of both formal and informal care practices for their family member. A gender balanced team of 4 data collectors (2 per district) conducted the FGDs. Data collectors were university graduates of Social Work and mental health nurses proficient in the Luganda language. Data collection was directly supervised by a masters degree holder with proven competence in qualitative methods of data collection and analysis who was in turn overseen by one of the co-authors. Transcription of audio-recorded data and its translation into the English language were done by the data collection team. District teams exchanged field notes and audio recordings of FGDs and worked with each others data sets to minimize biasing the process. Field notes were merged with transcriptions of audio data to generate complete records of the respective FGDs for further processes.

Data entry, coding and analysis were done by one of the co-authors supported by the other co-authors, using the Atlas.ti computer software. Analysis followed the framework approach whereby codes for anticipated content and themes, informed by four of the seven objectives of the main study and literature, guided data exploration [19]. Four objectives of the main study therefore, constituted the predetermined themes during analysis. The said objectives concerned: availability and accessibility of mental health services; family and community mental health seeking behaviours and illness care practices; perceived attitudes and support of community members towards persons with mental illness; and nature of relationships in families of persons with mental illness and their impact on family members. Several iterations of reviews and refinement of themes identified and confirmed coherent patterns until saturation. Three major themes freely emerged from the analysis namely: direct and practical care responses; emotional relationships and care; and the social and cultural context of care. Subsequent reviews identified and prioritised salient phrases and quotations on emerging themes.

## Results

From the focus group data, a kaleidoscopic picture emerges of the nature of care and caring in the context of severe mental illness for these families in the study. Whilst it was mostly family members who were caregivers, (i.e., grandparent, mother-in-law, cousin, brother or sister, husband, or wife) this group also included friends engaged in a care relationship. Thus, the term family care as used in this paper, reflects different types of family structures, bonds and connections, as established in a wider literature [6, 20]. The common denominator is the caregiver was a person in the life/network of the PLSMI willing and able to provide immediate, post discharge home based care. The results presentation is structured by the above-said three meta themes. The responses in the data include experiences across time, both before their family member was admitted to BNRMH, and prior recollections of post discharge care.

### Direct and practical care

Each of the caregivers in the focus group discussions had a relative or close friend on the convalescent ward at BNRMH. It was clear that beliefs that caregivers hold about mental illness influenced the form of direct care and support. This includes the belief voiced by caregivers in this study that mental illness is caused by traditional causes namely, evil spirits and witchcraft. The strength of this belief system is captured in the caregiver responses below.

*I think it is very rare for someone with such an illness to seek medical attention in the first place and this is because most people strongly believe in traditional causes.* (FGD, caregivers, Wakiso)

*I dont think it is common for people to go to the hospital when mental illness happens. Even me personally, on the first onset of mental illness in my patient, I did not think of hospital; we first locked him up for some time and did everything there. Then we invited the Imam (Muslim cleric) to pray for him and he improved for almost a year. But when he broke down again, we decided to come to the hospital.*(FGD, caregivers, Kampala)

*Since mental illness may not heal easily, it needs a strong will for someone to stick to medical care, resist the temptation for going to shrines, and worship centres. But because mental illness is not well understood, people try whatever they think can heal them or whatever other lay people advise them to do.* (FGD, caregivers, Kampala)

The caregiver role in supporting a person living with SMI is multi-dimensional. Carers discuss how they are active in supporting adherence to treatment; playing a role in monitoring treatment outcomes and possible medication side effects; and reinforcing psychosocial support and calming down aggression and challenging behaviour. Other aspects of practical care included providing food and shelter to meet basic needs. Maintaining productivity of the family member emerged as a theme in the context of low family resources. Findings revealed that some families heeded the advice of health workers by engaging the person in recovery in productive activities during periods of symptom remission, to ensure retainment and enhancement of their social skills and to promote and preserve their self-esteem. These include income generating activities that also enhance a sense of identity and being active members of the community.

Respondents noted the caring difficulties presented by the nature of symptoms that accompany some illnesses. Some symptoms, such as agitation and aggression, are difficult to manage if medication is not easily accessible. It was not atypical to hear this kind of response Sometimes, we take the patients to the health centres but they return untreated because the medicine is out of stock. This is made more challenging in the absence of transportation and access to health care supports. Some respondents shared the experience of often failing to find public means of transport to get to health facilities, citing instances where patients with active mental illness symptoms either face outright rejection by public transport operators, or exorbitant fare charges. This is made worse if there is not adequate money to meet transport fares on the part of the person with SMI and family member. These themes are conveyed in the extract below.

*Usually, no one wants to transport the mentally ill person because of fear of possible violence and destruction of the vehicleSometimes, due to the lengthy periods of medication, all the money is spent on treatment, leaving no money to transport the patient to the hospital for review or in case of a relapse*. (FGD of caregivers from Wakiso)

*My patient is usually given medicine when he is being discharged. My duty is to make sure he takes the medicine properly and I also watch out for side effects and take appropriate action In case the medicine is not effective, I am supposed to go report back to hospital I need to understand how they are supposed to feel and act accordingly.* (FGD of caregivers from Kampala)

*I wish there was medication that can enable the patient to stay at home for longer periods; say medicines that can be administered on a monthly basis. Currently, these patients often get tired of the medicines; they stop swallowing them, get re-admitted to hospital almost immediately.* (FGD of caregivers from Kampala)

A further common theme across the focus group discussions was the role the caregiver played in managing challenging behaviours. The following responses tell snippets of caregivers stories of the person they care for being violent and aggressive. The first respondent talks about the multidirectional impacts of this violence, for carers and communities and the person being cared for, who may face violence from the wider community. Damage and fear can arise for the family and community. The theme of fear and tension is highlighted in the third quote below.

*The patients are violent; they fight, destroy things and even beat people. In the end, you spend money replacing the destroyed things or even seeking treatment for injuries caused by the patient. They sometimes threaten the people at home and that is why some people tie them up. They can rape children in the community and this can be a very big problem because people can react by killing the patient.* (FGD of caregivers from Kampala)

*When I brought the patient to hospital, he was so deadly; he would beat us Sometimes you find him chasing and beating other people One time he beat our mother and he knows she is his mother*. (FGD of caregivers from Kampala)

*There can be a lot of fear and tension in the family due to violence (by their mentally ill member). When such things happen, the usual option is to alert the nearby authority like the Police who come and handcuff or shackle the patient, and then transport him or her to the hospital* (FGD of caregivers from Kampala)

Set against these challenging behaviours, some caregivers indicated their key role in supervising and protecting the family member from harm. In some cases, they also protected other people and property from a patients aggression. The dangers to the person with SMI are again apparent, with mentions of violent and abusive responses to them. The caregiver quoted below provides a picture of the many forms this abuse can take. From their perspective, this abuse stems from held views that a person with a mental illness is cursed.

*These patients suffer a lot; they are tied with ropes which cause wounds on their hands. They are beaten and others end up locked in specific rooms. Those who are taken to witch doctors are beaten there as a way of casting out the demons that they believe to possess the patients. Community members normally do not like being near them; they chase them away and sometimes even beat them up.* (FGD, caregivers, Kampala)

Watchfulness as a helping practice is evident in the following extracts. Knowledge about mental illness had helped them understand their family members illness; they now watch out keenly for the early warning signs and symptoms of relapse and they are able to take the patient to a health facility before the patient completely regresses. Acceptance, understanding and reinforcing of the family members value were also important components of care for one respondent, as seen in the first extract below.

*What the patient needs from us, the family, is to be there for them, provide each and everything they need no matter the cost. When they do wrong things when they are not in their right states of mind, we should be understanding and supportive, talk to them and make them feel they are still alive and valued.* (FGD of caregivers from Kampala)

*My patient doesnt break down abruptly; she normally shows signs that she is about to break down. So, as soon as we see that, we arrange to take her to hospital.* (FGD of caregivers from Kampala)

### Relationships and care

Findings revealed that in some families where the members accept the reality of the mental illness of their relative, there was a tendency to unite in support of their affected family member. The focus was on securing healing and wellbeing of their family member. As the two excerpts below make plain, there was a perception that a good relationship in the family can foster physical and emotional support that is important to the health outcomes of the person living with mental illness.

*I will talk about the positive part; we now have a good relationship as members of a family. I think it (good relationship) can positively affect the recovery of the patient because if the relationship is bad and the patient is mentioned in quarrels of family members, the patient thinks they are not loved.* (FGD, caregivers, Wakiso).

*...The relationship is good; actually, it has even become much better ever since my child became sick. My husband really cares about the child; he makes sure he gives all the support that the child and I need. We previously had many conflicts but they are not that many now; I can even say we are still in a relationship now because of the child.* (FGD, caregivers, Kampala)

To this effect, the family circumstances and mental illness has supported a positive bonding effect among some families as members stand together, as a unit, to provide care and support to the family member with mental illness. The above notwithstanding, perceptions of such cases where mental illness resulted in positive impacts on family relationships and bonding were less prominent.

For a majority/ great many of caregivers they recounted negative experiences of the effect of mental illness on family relationships. Perspectives emerged suggesting strained family relationships. There were some instances of family heads reportedly abandoning their families as they became overwhelmed with the intensity of required care; an absence of convincing explanation for the occurrence of the illness; and sometime the cost associated with its management. As noted earlier, violence was a recurring theme in the discussions.

*The relationship was previously good; but the patient [my sister] started getting misunderstandings with her husband. There was a lot of violence in the family; many meetings held to help them did not avail successful solutions until they separated Now even most of her relatives do not support her* (FGD, caregivers, Kampala)

In some cases, these strained relationships followed disagreements about help-seeking strategies with dissenting views around whether to seek medical care in hospital, or alternative non-medical options, or a combination of both. There was a general agreement that persons with mental illness also are negatively affected by these family dynamics, especially amidst conflict focused on the mental illness, and when there is open negativity about the person or circumstances of their condition. The following respondent captures this in their comments about their own family challenges.

*I would say the relationships are normally not very good when there is a mental patient/person with mental illness especially if the family already has other problems like poverty I would say it has been really challenging in our family.* (FGD, caregivers, Kampala)

### The social and cultural context of care

That care practices cannot be considered outside a wider social, economic and cultural context was evident in the focus group data. The wider contexts of community, social determinants of mental illness and culture both supported and presented challenges for the family. Persons with mental health disorders are often discriminated and rejected in their communities. Because of the negative attitude of the community towards them, as some respondents recounted, family with a mentally ill person do not freely mix with others in the community; they are often judged, avoided and isolated. These attitudes are apparent in the extracts below.

*The attitude of community members is negative; they isolate mentally ill persons and stigmatise them. No one can marry or get married to a person they know has mental illness.* (FGD of caregivers from Kampala)

*People need to be sensitised about mental illness so that they do not stigmatise us (family members) and also stop judging us that we are responsible for our patients conditions. It is very disturbing when one judges you wrongly, yet some of us really love our patients.* (FGD of caregivers from Kampala)

Analysis of the data indicates an element of community level fear when there is a person with mental illness in their community, especially when the patient may be violent. People fear that the person can harm them. Community members negative reactions are themes mentioned in the following responses:

*Most people believe that mental illness is a cultural issue and not an illness. So, they attribute it to witchcraft and many of them dont like to associate or get into contact with the affected people because they think they can contract the disease that way.* (FGD, caregivers, Wakiso)

*Community members sometimes beat our patients. aggressive patients; they can rape women the women are sometimes compelled to go back home early due to fear of being raped by mentally ill persons... Children are also always fearful.* (FGD of caregivers from Kampala)

*Community members attitude is very negative; people often run away from them and call them all sorts of names like, mad people Some people even beat them up and give them bad food because they consider them as mad. It is a growing culture because even children do the same things when they meet these patients on the way* (FGD, caregivers, Kampala)

There were also accounts where families of persons with severe mental illness experience multiple losses, one of which is the loss of active labour. The family loses not only the labour of the sick person when he/she is unwell but also that of the caregiver who spends much time caring for the patient. It was noted that it is generally more difficult to take care of people living with SMI compared to those with other types of illnesses because of levels of functioning. This situation is likely to strain the household resources.

*I dont have any other person who can help me to take care of the child. I cant work to get some money and all the others go to school The good thing now is that the students are at home for holidays, otherwise I would not be able to attend this discussion. *(FGD of caregivers from Kampala)

Respondents observed that women can be blamed for mental illness in the family. This blame was couched in views that cause of the condition is genetic and from the mothers side of the family, or a view that the husband is not the biological father. Perceived disagreements due to family members suspecting curses or genetic links to mental illness was a manifest theme. In the extract below, a caregiver tells a story of a father who feared mental illness was inherited and because of this, left his children and wife. Other community members recounted known examples where the incidence of mental illness had created family tension and breakdown.

*There is a family I know; the husband separated with his wife because the wife went to attend to her mentally ill relative in the hospital. The man believed that the woman might have mental illness genes; he feared his children with her might get the same problem so he just left like that.* (FGD, caregivers, Wakiso)

Although there is a dominant convergence of views demonstrating negative attitudes towards persons with mental illness, there was also mention of positive and supportive attitudes and behaviours, captured in the following quote from a caregiver.

*there are some people who also show supportive attitudes, like giving food to the patients and even accommodating them* (FGD, caregivers, Kampala)

Places of worship were social support systems and networks of care for people with mental illness. Considering this, some caregivers noted how these religious based networks are potentially good resources to tap into, to support patients at the community level. They nevertheless recognised that some religions preach beliefs that potentially hinder adherence to medications provided in the health facilities. The cooperation of the churches was advocated by a caregiver, as seen below.

*We need cooperation of the churches. Since I know the church to which my daughter goes, I will approach and ask them to encourage her to take her medicine even as she prays because whenever she is discharged, she goes to church and stops taking medicine In one week, she relapses.* (FGD of caregivers from Kampala)

## Discussion

This research exploring family care practices for people living with SMI is from a baseline study conducted in Uganda to inform the work of the NGO YouBelong Uganda. The purpose in doing the wider baseline study was to generate knowledge to help craft effective community-based hospital discharge and post discharge processes for people living with SMI. SMI is a global health problem [20]. The public health implications of SMI in sub-Saharan Africa are increasing within a system with limited community-based infrastructure to meet peoples needs [9, 21,24]. In this context, family care is a key resource for mental health recovery [24]. In the wider literature, family care for people living with SMI is linked to both support to mental health recovery and as a trigger for preventable mental hospital readmissions. Family characteristics, access to material resources, beliefs, levels of criticism (EE) towards the person living with SMI and past experiences, have a bearing on care experiences for both the person living with SMI and family members [7, 24].

Keating et al., posit that family care as a life course domain has components of both doing tasks and being in relationship [6, 25]. In recounting their experiences, the caregivers involved in this research describe both care tasks and care relationships. Family include kinship relationships and friendships; relationships that change over time. These caregivers undertook practical care tasks; providing housing, food, positive reassurance, watching for signs of relapse, ensuring medication is taken and supporting their family member in productive activity, which can include income generation. We can add to this listing the relationship work Keating et al., [6] conceptualise, and managing substantial challenges. Supporting the management of active symptoms of psychotic illness, like agitation and aggression, particularly if medication is in short supply, dealing with the day-to-day realities of internalised and external social stigma and discrimination, and coping with and managing family conflict are components of what respondents describe as family care. The extent of care tasks draws attention to the absence of other assistance and the burden of care [24]. Furthermore, the interpersonal relationships that are within the dynamics of family life and caring are powerfully conveyed in the respondents emotional stories. Care practices are influenced by these relationships, together with social and cultural dynamics, realities of low incomes [24] and the cumulative effects of the events of a life course [6, 25]. The interplay between care relationships and care tasks is apparent.

Fear, violence and community rejection and hostility towards the family member with SMI and the caregivers feature in the focus group narratives. This accords with the research literature on stigma and discrimination in the context of mental illness [13, 14, 26, 27]. Several family carers in the study are dealing with a community perception that the patient and his/her family, and often the women, are responsible for the mental illness. This community attitude is rooted in traditional beliefs and practices about the causes of mental illness [21]. As Molodynski et al. [21. p.98] write Traditional beliefs regarding causation mean that many people with mental health disorders do not present for treatment. The use of traditional and faith healers is a first port of call for an overwhelming number of families as they try to make sense of their family members state of mind and behaviours. Some of the factors influencing care relationships and experiences of caring, are in the control of the family system whilst others are clearly not, as understandings about the social determinants of mental health indicate [3]. It seemed that some family carers walk a precarious tightrope as they undertake tasks, maintain relationships, manage violence and hostility, and the challenges that are inherent in practical care.

The findings from this research have implications for mental health service design and delivery of interventions to support family caregivers of people living with SMI. As Addo et al. [24] write, there is an urgent need to translate research about family carer burdens in sub-Saharan Africa, into workable interventions that will support both people living with SMI and carers. From the understandings gained in this study, implementing practical family-oriented interventions in the context of post hospital support for people living with SMI, necessitates using a family orientated model that recognises the complexity, messiness and challenges family care givers face. As the data in this study show, the family care environment is dynamic, in a context where stigma about mental illness impacts everyday living, and health system resources are scarce. Responsive interventions need to balance the reality of high use of traditional and faith healers, alongside evidence based mental health care. While cultures adapt, change and develop over time, the question needs to be asked as to how evidence based psychosocial counselling and support in post discharge interventions, together with approved pharmacological medications, can fit with such a cultural interpretation of the cause and the cure of mental illness? Whatever answer is given to the question, a tension between two quite different cultural understanding is highly likely to remain, and such tension would need to be held with sensitivity and a context of ongoing dialogue.

This calls for a complex approach to the provision of effective support for families in their support for someone living with SMI [2]. Complex can be a term that is used loosely, but what the findings of this study suggest is the importance of models of hospital discharge practice that take into consideration the multiple factors interacting to shape a dynamic family care environment, as identified by Molodynski and colleagues [21] and Dirik et al. in their work on family involvement in mental health care delivery [1]. This practice should facilitate both person and family centred responses, and in ways that are adaptive to diverse families in different, and often difficult situations. This is especially so for those living in a low-income context, with limited (mental) health system infrastructure, limited access to medications and practical impediments to access services, such as poor transport, and low material resources [2, 24]. The family, in some form, becomes a daily resource for mental health support, and providing emotional support, a sense of belonging, and basic care, becomes the foundation upon which to build further health services supports, notwithstanding the social, cultural, and economic challenges faced by families [24].

There are limitations to the work presented in this paper. This is a report only of the subjective accounts of people who care for someone who is about to be discharged from the national mental hospital. It is a point in time for people who live in two districts in Uganda and who have family members as inpatients in the hospital. It does not include the crucial perspective of the family member who is living with SMI, and as well, how they care and support others in a family system. People living with SMI also care. There is a body of work which explores mutual caring relationships [1. p.7] [6, 25]. Further research could do more fine-grained inquiry especially to better understand trajectories of family care and support in the context of SMI, along the lines suggested by writers such as Keating et al. [6, 25].

## Conclusion

With limited resources to constitute Ugandas health system, reliance on the family as a unit, however family may be configured, becomes an imperative factor in the recovery of a person living with SMI. The Ugandan research reported in this paper shows the complex challenges facing carers in their support for a family member in recovery from SMI. These will be particular to the day-to-day realities of each family. Yet, there are common issues which impact on care in these contexts. These include the insidious negative effects of stigma and discrimination about SMI on care, challenging behaviours exacerbated by poor medication access, lack of services and economic challenges. These do not negate the strengths and emotional solidarity within interpersonal caring relationships, but they can place these relationships under tremendous strain. Economic empowerment and empowering family carers, the person living with SMI and the wider community with knowledge about causes and treatment of SMI, mental health and the recovery process, as well as building social support capabilities, should be the necessary threads in an approach that recognises the family as a key resource and an active agent in a family members mental health recovery.

## Data Availability

The data that support the findings of this study are available from YouBelong Uganda but restrictions apply to the availability of these data, which were used under license for the current study, and so are not publicly available. Data are however available from the authors upon reasonable request and with permission of YouBelong Uganda.

## Abbreviations

BNRMH: Butabika National Referral Mental Hospital
YBH: YouBelong Home
YBU: YouBelong Uganda
SMI: Severe Mental Illness
EE: Expressed Emotion
